# Correction: Aksic et al. The Neuroprotective Effect of Neural Cell Adhesion Molecule L1 in the Hippocampus of Aged Alzheimer’s Disease Model Mice. *Biomedicines* 2024, *12*, 1726

**DOI:** 10.3390/biomedicines13020290

**Published:** 2025-01-24

**Authors:** Miljana Aksic, Igor Jakovcevski, Mohammad I. K. Hamad, Vladimir Jakovljevic, Sanja Stankovic, Maja Vulovic

**Affiliations:** 1 Center for Medical Biochemistry, University Clinical Center of Serbia, 11000 Belgrade, Serbia; miljanaaksic@gmail.com (M.A.); sanjast2013@gmail.com (S.S.); 2 Institut für Anatomie und Klinische Morphologie, Universität Witten/Herdecke, 58455 Witten, Germany; 3 Department of Neuroanatomy and Molecular Brain Research, Institute of Anatomy, Ruhr Universität Bochum, 44801 Bochum, Germany; 4 Department of Anatomy, College of Medicine and Health Sciences, United Arab Emirates University, Al Ain P.O. Box 64141, United Arab Emirates; 5 Center of Excellence for Redox Balance Research, Cardiovascular and Metabolic Disorders, Department of Physiology, Faculty of Medical Sciences, University of Kragujevac, 34000 Kragujevac, Serbia; drvladakgbg@yahoo.com; 6 Faculty of Medical Sciences, University of Kragujevac, 34000 Kragujevac, Serbia; 7 Department of Anatomy, Faculty of Medical Sciences, University of Kragujevac, 34000 Kragujevac, Serbia; maja@medf.kg.ac.rs

There was an error in the original publication [1]. The animal protocol reference was mistakenly incomplete.

## Text Correction

A correction has been made to 2. Materials and Methods, 2.1. Mice, Paragraph 1, as follows:

“All experiments were conducted in accordance with the “Principles of laboratory animal care” (NIH publication No. 86-23, revised 1985), as well as with German and European Community laws on the protection of experimental animals. The experiments were approved by the responsible committee of the State of Hamburg (permit number 09/098) and by the Ethics Committee of the University of Kragujevac (permit number 01-617/4) and were conducted in association with our supervising veterinarian Dr. Andreas Haemisch.” 

## Institutional Review Board Statement Correction

A correction has been made to Institutional Review Board Statement, as follows:

“The experiments were approved by the responsible committee of the State of Hamburg (permit number 09/098) and by the Ethics Committee of the University of Kragujevac (permit number 01-617/4) and were conducted in association with our supervising veterinarian Dr. Andreas Haemisch.” 

The authors state that the scientific conclusions are unaffected. This correction was approved by the Academic Editor. The original publication has also been updated.
