# Supplementary material for: The Neuroprotective Effect of Neural Cell Adhesion Molecule L1 in the Hippocampus of Aged Alzheimer’s Disease Model Mice
Source: Biomedicines. 2024 Aug 1;12(8):1726. doi: 10.3390/biomedicines12081726 (PMC11351965; doi:10.3390/biomedicines12081726)
Supplement: Supplementary file 1 [file biomedicines-12-01726-s001.zip › biomedicines-3077312-supplementary.pdf]

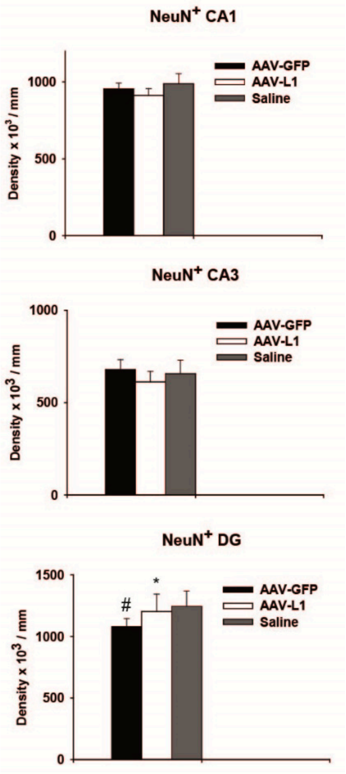

Supplementary Figure S1. Saline solution injection control. Densities of hippocampal NeuN-positive neurons in the pyramidal layer of the CA1 (B), CA3 (D) and granule cells in the DG (F) in saline injected wild-type mice (Saline), and AAV-L1, AAV-GFP injected APP/PS1 mice. Data are shown as mean + standard deviation. Asterisk indicates difference between treatments, hashtag indicates difference from the saline injected control, one-way ANOVA with Holm-Sidak posthoc, $p < 0.05$; n = 5 mice/group
